# MetAMOS: a metagenomic assembly and analysis pipeline for AMOS

**DOI:** 10.1186/gb-2011-12-s1-p25

**Published:** 2011-09-19

**Authors:** Todd J Treangen, Sergey Koren, Irina Astrovskaya, Dan Sommer, Bo Liu, Mihai Pop

**Affiliations:** 1 Center for Bioinformatics and Computational Biology, University of Maryland, College Park, MD 20742, USA; 2 The McKusick-Nathans Institute for Genetic Medicine, The Johns Hopkins University School of Medicine, Baltimore, MD 21205, USA; 3 Department of Computer Science, University of Maryland, College Park, MD 20742, USA

## Background

Metagenomics has opened the door to unprecedented comparative and ecological studies of microbial communities, ranging from the sea [1] to the soil (the terragenome) to within the human body [2,3]. Most analyses begin with assembly, as the short reads that are characteristic of most datasets severely limit the ability to classify the data taxonomically [4-7] and require considerable computational resources to perform comparative analyses (such as BLAST against public databases). In addition, given that many sequences are likely to be from novel organisms, classification methods relying on databases fail to acknowledge most of the novel species present in the dataset. In an attempt to move away from reference-based analysis, computational tools based on promising algorithmic and statistical methods for metagenomic *de novo* assembly have recently started to emerge [8,9]. However, to date, they either are ill-suited to large datasets or have yet to offer significant improvements over existing genome assemblers that were not designed for metagenomic assembly.

## Methods

Here, we describe MetAMOS [10], an open-source, modular assembly pipeline built upon AMOS and tailored specifically for metagenomic next-generation sequencing data. MetAMOS is the first step toward a fully automated assembly and analysis pipeline, from mated reads (Illumina and 454) to scaffolds and ORFs. Currently, MetAMOS has support for four assemblers (SOAPdenovo [11], Newbler, CABOG and Minimus [12]), three annotation methods (BLAST, PhymmBL and MetaPhyler), two metagenomic gene prediction tools (MetaGeneMark and Glimmer-MG) and one unitig scaffolder engineered specifically for metagenomic data (Bambus 2). We also provide a novel graph-based algorithm to propagate annotations rapidly to all contigs in an assembly using, for example, only the largest contigs or contigs with high-confidence classification. MetAMOS has three principal outputs: subdirectories containing FASTA sequence of the contigs/scaffolds/ variant motifs belonging to a specified taxonomic level, a collection of all unclassified/potentially novel contigs contained in the assembly, and an HTML report with detailed assembly statistics and summary charts.

## Results and conclusions

We compared MetAMOS with other metagenomic assembly tools (Meta-IDBA and Genovo) and with genome assemblers that have previously been used with metagenomic data (CA-met and SOAPdenovo). We used both a mock/artificial dataset generated for the Human Microbiome Project (HMP) project and real metagenomic samples from the HMP and its European counterpart (MetaHIT). On the mock dataset, MetAMOS compares favorably to existing metagenomic and genomic assemblers with respect to several validation metrics that take into account contig accuracy in addition to size. On the real dataset, MetAMOS also outperforms the existing software. These improvements can largely be attributed to heavy reliance on Bambus 2 and to assembly verification techniques that help identify and remove potentially chimeric contigs while running the pipeline.

In terms of biology, we were able to report several novel variant motifs that would be challenging at best to identify and extract from the output of other methods. In addition, much emphasis was placed on making MetAMOS compatible with a variety of next-generation sequencing technologies, genome assemblers and annotation methods, making the pipeline highly customizable for the beginner and advanced bioinformatics user alike.
